# Capacities of Migrating CD1b^+^ Lymph Dendritic Cells to Present *Salmonella* Antigens to Naive T Cells

**DOI:** 10.1371/journal.pone.0030430

**Published:** 2012-01-18

**Authors:** Michel Olivier, Benjamin Foret, Yves Le Vern, Laurence A. Guilloteau

**Affiliations:** UR1282 Infectiologie Animale et Santé Publique, Institut National de la Recherche Agronomique, Nouzilly, France; Institut Pasteur, France

## Abstract

Dendritic cells (DCs) are well known as professional antigen-presenting cells (APC) able to initiate specific T-cell responses to pathogens in lymph nodes (LN) draining the site of infection. However, the respective contribution of migratory and LN-resident DCs in this process remains unclear. As DC subsets represent important targets for vaccination strategies, more precise knowledge of DC subsets able to present vaccine antigens to T cells efficiently is required. To investigate the capacities of DCs migrating in the lymph (L-DCs) to initiate a specific T-cell response, we used physiologically generated DCs collected from a pseudoafferent lymphatic cannulation model in sheep. The CD1b^+^ L-DCs were assessed for presenting antigens from the vaccine attenuated strain of *Salmonella enterica* serovar Abortusovis. CD1b^+^ L-DCs were able to phagocytose, process and to present efficiently *Salmonella* antigens to effector/memory T cells *in vitro*. They were shown to be efficient APC for the priming of allogeneic naive T cells associated with inducing both IFN-γ and IL-4 responses. They were also efficient in presenting *Salmonella* antigens to autologous naive T cells associated with inducing both IFN-γ and IL-10 responses. The capacities of L-DCs to process and present *Salmonella* antigens to T cells were investigated *in vivo* after conjunctival inoculation of *Salmonella*. The CD1b^+^ L-DCs collected after inoculation were able to induce the proliferative response of CD4^+^ T cells suggesting the *in vivo* capture of *Salmonella* antigens by the CD1b^+^ L-DCs, and their potential to present them directly to CD4^+^ T cells. In this study, CD1b^+^ L-DCs present potential characteristics of APC to initiate by themselves T cell priming in the LN. They could be used as target cells for driving immune activation in vaccinal strategies.

## Introduction

Dendritic cells (DCs) are well known as professional antigen-presenting cells (APC) able to initiate specific T-cell responses to pathogens in lymph nodes (LN) draining the site of infection. However, the respective contribution of migratory and LN-resident DCs in this process remains unclear [1]. Moreover, the understanding of this complex process depends on the different DC subsets described. In mice, there are migratory DC subsets including epidermal Langerhans cells (LCs), CD11b^+^ CD103^−^ and CD11b^−^ CD103^+^ dermal DCs, LN-resident DCs comprising both CD8α^+^ and CD8α^−^ DCs, and inflammatory DCs recruited during infection [2], [3]. Studies based on experimental mouse models of cutaneous infection support the role of LCs in antigen (Ag) transport to the peripheral LN but not directly in the induction of pathogen-specific T-cell responses [4], [5]. Numerous studies examining the role of migratory and LN-resident DCs in the induction of CD8^+^ T cell-mediated immunity to viruses after cutaneous infection have shown the exclusive cross-presentation of Ag by CD8α^+^ DCs resident in LN, and the role of migratory DCs in delivering and transferring Ag to resident CD8α^+^ DCs [3]. However, these conclusions may not be applicable to the priming of cytotoxic T-lymphocyte (CTL) responses to all viruses since migratory skin DCs have been shown to present lentivirus-derived ovalbumin (OVA) directly to LN CD8^+^ T cells [6], or at least in cooperation with LN-resident DCs [7]. Moreover, dermal migratory DCs have been shown to play a role in generating CD4^+^ T-cell responses following subcutaneous (SC) influenza infection [8]. Regarding the LN-resident DCs, CD8α^−^ DCs seem to be more efficient than CD8α^+^ DCs at presenting exogenous antigens by MHCII molecules [9]. For *Salmonella*, the involvement of both CD8α^+^ and CD8α^−^ splenic DCs in the priming of T cells was reported in mice, but the involvement of migratory DC subsets was not investigated [10]. The respective contribution of migratory and LN-resident DCs in T-cell priming is thus dependent on the pathogen encountered, but also influenced by the *in vivo* infection route. Herpes simplex virus (HSV) is rapidly presented by LN-resident DCs [11] or dermal DCs [12] to CD4^+^ and CD8^+^ T cells after SC injection, whereas HSV Ag is mainly presented by migratory DCs after mucosal administration [11].

Most of these studies were performed in mouse models using diverse experimental strategies. Although these models assessed in detail the diverse functions of migratory DC subsets isolated from tissues, they did not investigate them in the draining lymph directly before their arrival in the LN. As DCs are important targets for vaccination strategies, more precise knowledge of DC subsets able to present vaccine antigens to T cells efficiently is required. Moreover, attenuated pathogens such as *Salmonella* can be attractive as a vehicle to deliver Ag to the appropriate DCs involved in a protective immune response.

These questions can be investigated further in large animals, using physiologically generated DCs collected from a pseudoafferent lymphatic cannulation model [13], [14]. The ruminant lymph DCs (L-DCs) were originally defined on the expression of the CD1b and CD14 molecules [15]. Several studies have investigated and showed the capacities of L-DCs to acquire soluble antigen *in vitro* or *in vivo* and to present it directly and specifically to autologous T cells [14]. More recent studies have described different L-DC subsets including plasmacytoid [16] and CD8α-like DCs [17]. However, a few studies have analyzed the interaction between DCs and *Salmonella*, and their involvement in the priming of naive T cells *in vivo* [10]. An *in vitro* study showed that fewer *Salmonellae* were taken up by CD1b^+^ L-DCs than by monocyte-derived macrophages [18]. Moreover, one of our *in vivo* studies showed that CD1b^+^ L-DCs did not play a major role in *Salmonella* transport to LN after SC infection of the upper respiratory tract with a live vaccine, despite an increased flow of these cells in the lymph [19]. This study showed the predominant role of neutrophils in the the live vaccine uptake as it was also showed in another study using particulate antigen [20]. This approach can assess directly the ability of migratory L-DC subsets to perform Ag presentation at steady state and under infectious conditions.

The aim of this study was to investigate in detail the capacity of sheep CD1b^+^ L-DCs, collected from afferent lymph draining the skin or the head mucosae, to present *Salmonella* antigens to T cells *in vitro* and *in vivo* after mucosal administration. To this end, we used the vaccine-attenuated strain of *Salmonella* demonstrated to induce protection against abortive salmonellosis in sheep [21], and previously used to assess the capacity of L-DCs to uptake and transport *Salmonella* to LN [19].

## Materials and Methods

### Ethics statement

The animal experiments were conducted under a license issued by the Direction des Services Vétérinaires of Tours (accreditation B-37-175-3) and were approved by the Regional Centre-Limousin Ethics Committee (CL2006-012).

### Sheep and surgery

‘Prealpes du sud’ ewes (one to four years old) originating from the Unité Commune d'Expérimentation Animale (INRA, Jouy-en-Josas, France) or from the Plateforme d'Infectiologie Expérimentale (PFIE) (INRA, Nouzilly, France), were housed in the PFIE for surgery and *Salmonella* infection. They were born and raised in salmonellosis-free herds. Prescapular lymph duct cannulation was performed on the right side of the sheep to collect pseudo-afferent lymph from skin as described previously [13], and cervical lymph duct cannulation was performed on the left side of the same sheep, to collect lymph from the head mucosae as described previously [22]. A total of 11 sheep were used for the study, of which eight were successfully cannulated. Sheep received a daily subcutaneous (SC) injection of calcium heparin (300U per kg per day) (Calciparine®, Sanofi-Avantis, France).

### Collection of lymph and lymph cell storage

Lymph was collected continuously in 250 ml sterile polypropylene flasks containing 10 ml of buffered saline supplemented with antibiotics (100 IU/ml of penicillin and 100 µg/ml of streptomycin, Sigma-Aldrich, St Louis, MO) and sodium heparin (250 IU/ml) (héparine choay®, Sanofi-Avantis, France) to prevent both bacterial contamination and clotting. Flasks were changed twice a day, the lymph volume was determined and the viable cell number was assessed using the trypan blue dye exclusion test. Lymph cells were spun down at 400 *g* for five min, step-frozen in fetal calf serum (FCS) containing 10% dimethyl sulfoxide, and kept in liquid nitrogen. All the analyses described hereafter were performed on lymph cells from frozen samples. Over 95% viability was obtained after thawing.

### Phenotypic analysis of lymph DC cells (L-DCs) by flow cytometry

Lymph cells were thawed, washed once in HANK'S Balanced Salts (HBSS) medium supplemented with 2.5% FCS, 20 IU/ml of penicillin and 20 µg/ml of streptomycin (HBSS–FCS) and adjusted to 5×10^6^ cells/ml. Labelling was performed in 96 round-bottom well microplates (BD Falcon™, Becton Dickinson, Franklin Lakes, NJ) using 5×10^5^ cells per well. Cells were incubated with 50 µl of antibodies diluted at optimal concentrations in RPMI 1640 medium (Life Technologies, Cergy Pontoise, France) supplemented with 10% FCS, 2 mM L-glutamine, 1mM sodium pyruvate, and 50 µM ß-mercaptoethanol, 20 IU/ml of penicillin and 20 µg/ml of streptomycin (complete medium) for 20 min on ice with gentle stirring, and washed three times with HBSS–FCS after each step.

Cells were first incubated with one of the primary or appropriate isotype control monoclonal antibodies (mAbs) at equivalent concentrations to primary mAbs (Table 1) and then with R-Phycoerythrin (RPE)-conjugated F(ab')_2_ fragment goat anti-mouse (GAM) IgG (1∶200) (Jackson ImmunoResearch, Suffolk, UK). Cells were further incubated with anti-CD1b mAb (Table 1) followed by FITC-conjugated GAM IgG2a (1∶200)(Caltag Laboratories, Burlingame, CA). After washes, cells were resuspended and fixed in 100 µl of 1% paraformaldehyde in buffered saline. Thirty to sixty thousand events were analysed with a FACSCalibur^TM^ (Becton Dickinson) using the CellQuestPro^TM^ software analysis programme (Becton Dickinson).

**Table 1:** Primary antibodies.

Specificity	Clone	Isotype	Designation	Source
Bovine CD1b	Th97A	IgG2a	Th97A	VMRD^a^
Ovine CD4	17D1	IgG1	17D1	VMRD
Ovine CD4	SBUT4	IgG1+IgG2a	SBUT4	Melbourne University
Ovine CD62L	DU1-29	IgG1	DU1-29	VMRD
Bovine CD8ß	CC58	IgG1	MCA1654	Serotec^b^
Bovine CD11b	MM12A	IgG1	MM12A	VMRD
Ovine CD11c	129F7	IgG1	OM1	M. Pépin^c^
Ovine CD14	VPM65	IgG1	VPM65	J. Hopkins^d^
Bovine CD26	CC69	IgG1	MCA1652	Serotec
Bovine CD40	ILA156	IgG1	ILA156	J. Hope^e^
Bovine CD44	BAT31A	IgG1	BAT31A	VMRD
Ovine CD45R	20.96	IgG1	MCA2221	Serotec
Bovine CD80	ILA159	IgG1	ILA159	J. Hope
Bovine CD86	ILA190	IgG1	ILA190	J. Hope
Bovine CD205	CC98	IgG2b	MCA1651	Serotec
Human CD206	ILA190	IgG1	ILA190	J. Hope
Human CD209	AHP627	Poly IgG	AHP627	Serotec
Bovine γδ TCR	86D	IgG1	86D	VMRD
Ovine MHCII	SBU2	IgG1	28.1	Serotec
Bovine SIRP-α	ILA24	IgG1	ILA-24	J. Hope
Isotype control	X0931	IgG1	X0931	DakoCytomation^f^
Isotype control	X0943	IgG2a	X0943	DakoCytomation

a VMRD, Pullman, WA.

b Serotec, Düsseldorf, Germany.

c M. Pépin, ENVL, France.

d J. Hopkins, University of Edinburgh, UK.

e J. Hope, IAH Compton, UK.

f DakoCytomation, Fort Collins, CO.

Cells were analysed in a cell population gated on the basis of forward and scattered angles. The CD1b^+^ L-DCs were selected with appropriate gating and analysed for the expression of different markers.

### L-DC subsets sorting using fluorescence-activated cell sorting

Lymph cells were quickly thawed and washed once in HBSS-FCS. To deplete lymphocytes, cells were first incubated with a mixture of mAbs including anti-ruminant CD4 (17D1), CD8, γδ TCR, CD45R (2 µg/ml of each mAb for 1×10^8^ cells) (Table 1) for 20 min on ice with gentle stirring, washed three times with HBSS–FCS followed by RPE-conjugated GAM IgG labelling. Cells were further incubated with anti- CD1b mAb, followed by FITC-conjugated GAM IgG2a. After three washes with HBSS–FCS, cells were resuspended in RPMI medium without FCS before sorting. Appropriate IgG1 and IgG2a isotypes (Table 1) were used at equivalent concentrations to the primary mAbs to produce controls. CD1b^+^ L-DCs were sorted using a fluorescence-activated cell sorter (MoFlo®, DakoCytomation, 4850 Innovation Drive, Fort Collins, CO) after gating on a population negative for lymphocyte markers and positive for CD1b. The proportion of the CD1b^+^ L-DCs subset was enriched from 1% in the lymph to 96% with an average purity of over 98%.

### RNA extractions and reverse transcriptase PCR (RT-PCR)

Messenger RNA was extracted from sorted CD1b^+^ L-DCs using the Dynabeads® mRNA DIRECT™ Micro Kit (Invitrogen Dynal AS, Oslo, Norway). The mRNA was processed for reverse transcription with MuMLV reverse transcriptase (25U/µl) (Eurogentec, Liège, Belgium) and Oligo-dT (133 pmole/µl) (Eurogentec). The reaction was maintained for 90 min at 37°C and then heat-inactivated at 85°C for 10 min. The generated cDNA was then analysed for the presence of sequences encoding for DC-SIGN, CD103, CCR7, glyceraldehydes-3-phosphate dehydrogenase (GAPDH) and hypoxanthine-guanine phosphoribosyltransferase (HPRT) by either end-point or real-time quantitative PCR. The cDNA control was produced as described above from ovine spleen cells stimulated by both LPS and Concanavalin A. Primers (Table 2) were designed using Clone Manager 9 (Scientific & Educational Software, Cary, NC) and purchased from Eurogentec. DNA was amplified with REDTaq™ DNA Polymerase (1U/µL) (D5684, Sigma-Aldrich) for 34 cycles at the appropriate annealing temperature for 60 s (Table 2) and at 72°C for 60 s. PCR products were run on 1% agarose gel with ethidium bromide staining.

**Table 2:** Primers used for PCR and real-time RT-PCR analysis.

Target mRNA	Primer sequence	Annealing temperature (°C)	PCR product (bp)	Accession number
CCR7	S:GGGAAGCCAATGAAGAAC	60	185	AY834253
	AS:TACATGATCGGGAGGAAC			
CD103	S:GACAGCCAAGACGTTAGAG	58	205	XM2695790
	AS:TGACAGTCGTGAGGTTGAG			
DC-SIGN	S: GAGACCGTGAGAACAACAG	58	206	EU431222
	AS:GGTCCAGGAGAAGAAGTAG			
GAPDH	S:GGCGTGAACCACGAGAAGTATAA	60	119	NM1190390
	AS: CCCTCCACGATGCCAAAGT			
HPRT	S: AAACCAAAGATGGTCAAGGT	56	200	BC103248
	AS: TCTTAGGCTTTGTATTTTGCTT			

Quantitative real-time PCR (qPCR) was carried out using diluted cDNA, in duplicate, with the IQ SYBR Green Supermix (Bio-Rad, Hercules, CA) following the manufacturer's recommendations. Cycling conditions were 95°C for five min, followed by 39 cycles with denaturation at 95°C for 10 s and the appropriate annealing temperature (Table 2) for 15 s. PCR reactions were run on a Bio-Rad iCycler iQ (Bio-Rad). The specificity of the qPCR reactions was assessed by analysing the melting curves of the products and size verification. Samples were normalized internally using the cycle quantification (Cq) of GAPDH and HPRT simultaneously as references in each sample. Cq values were extracted with the qPCR instrument software and subsequently imported into qbase^PLUS^ (http://www.qbaseplus.com) for quality control and generation of the standard curves. Relative quantities were calculated using the qBase quantification model which enables PCR efficiency correction, multiple reference assay normalization, proper error propagation and, if necessary, inter-run calibration [23].

### Lymphocyte subset sorting using immunomagnetic microbeads

Peripheral blood mononuclear cells (PBMC) were obtained from fresh blood diluted 1/2 in phosphate buffered saline without calcium and magnesium (PBS) and layered onto Histopaque® 1077 (density 1.077, Sigma-Aldrich). After centrifugation at 1600*g* for 20 min at room temperature, PBMC were harvested at the interface and washed three times in HBSS-FCS. For the positive selection of CD4^+^ or CD62L^+^ T cells, PBMC were then incubated with anti- CD4 (SBUT4) or anti- CD62L mAb (Table 1), washed three times in PBS supplemented with 0.5% bovine serum albumin (BSA, A7906, Sigma-Aldrich), and further incubated with GAM IgG (H+ L)-coated magnetic microbeads according to the manufacturer's recommendations (GAM IgG MACS® microbeads, 130-048-402, Miltenyi Biotec, Paris, France). After three washes, cells were positively selected with an MS column and octoMACS™ separator (Miltenyi Biotec), and the efficiency of the resulting positive selection was checked by flow cytometry after incubating the sorted cells with RPE-conjugated GAM IgG (Table 1). The purity of CD4^+^ T cells and CD62L^+^ T cells were on average over 96% and 90% respectively.

### Preparation of Salmonella and infection

The *Salmonella enterica* serovar Abortusovis Rv-6 strain is a live attenuated vaccinal strain described previously [19] and stored in aliquots at −80°C in 10% glycerol-buffered saline. Aliquots were thawed, washed in buffered saline and the bacterial suspension adjusted to the appropriate concentration for each experiment. For *in vivo* infection, sheep were inoculated either by the SC route behind each shoulder with 1 ml containing 0.5 to 0.8×10^9^ *Salmonella*, or by the conjunctival route with 1.2×10^9^ *Salmonella* administered in four drops of 50 µl in the left eye drained by the cannulated cervical duct. The administration of *Salmonella* by the conjunctival route was carried out after cervical cannulation, and lymph was collected for 72 hours for analysis. For the *in vitro* experiment with L-DCs, the bacterial suspension was adjusted to the appropriate concentration in complete medium without antibiotics. The GFP-*Salmonella* Rv-6 strain, obtained by transformation with the plasmid pBRD940 [18], was also used for phagocytosis assays. The purity of the *Salmonella* culture and the number of viable bacteria were checked by plating serial dilutions on tryptic soy agar (TSA, Difco Laboratories, Detroit, MI).

### Endocytosis and phagocytosis assays

FITC-conjugated ovalbumin (FITC-OVA, 0-23020, Molecular Probes®, Invitrogen) was used to examine the endocytic ability of the L-DCs. Lymph cells were adjusted to 1×10^6^ cells/ml of complete medium and incubated at 37°C or 4°C for one hour with 200 µL of FITC-OVA (50 µg/ml) [24]. Controls included cells incubated at 37°C and 4°C without FITC-OVA. For phagocytosis assays, lymph cells were infected by *Salmonella* (100 bacteria/cell) for 30 min at 37°C. Cells were then washed with medium supplemented with gentamicin (G1397, Sigma-Aldrich) once at 200 µg/ml and twice at 50 µg/ml to remove and kill any remaining extracellular bacteria. Cells were then washed with HBSS-FCS and first incubated with anti-CD1b mAb followed by RPE-conjugated GAM IgG (Table 1). After washes, cells were incubated with a mixture of mAbs including anti-ruminant CD4, CD8, γδ TCR, CD45R labelled with the Alexa Fluor® 647 using the monoclonal antibody labelling kit according to the manufacturer's recommendations (A-20186, Molecular Probes®, Invitrogen). After washes, cells were resuspended and fixed in 100 µl of 1% paraformaldehyde in buffered saline. Thirty thousand events were analysed using FACS. The CD1b^+^ L-DCs were selected with appropriate gating, and the proportion of FITC labelled cells was analysed.

### Antigen presentation assays by L-DCs

To assess the allogeneic reaction, sorted CD1b^+^ L-DCs were resuspended in complete medium and seeded in triplicates (100 µl/well) at different ratios in round-bottom plates (Falcon 3077, Becton Dickinson) for 24h at 37°C. Purified allogeneic CD4^+^ or CD62L^+^ T cells (1×10^5^/100 µl/well) were then added to CD1b^+^ L-DCs. After 72h, 150 µl of supernatant were harvested and frozen at –80°C for cytokine detection, and 150 µl of fresh complete medium were added. The co-cultures were further incubated for 72h and proliferation was assessed by [^3^H]-thymidine incorporation (1 µCi/3.7×10^4^ Bq, NEN Research Products, Paris, France) for the last eight hours of culture followed by scintillation counting (Packard 1600TR meter, Meriden, CT).

To assess *Salmonella* presentation, CD1b^+^ L-DCs were resuspended in complete medium without antibiotics, distributed in round-bottom plates (1×10^3^/well) and infected by *Salmonella* as described above. After washes with antibiotics to remove and kill any remaining extracellular bacteria, cells were resuspended in complete medium supplemented with gentamicin (50 µg/ml) (100 µl) and incubated for 24h at 37°C. Autologous purified T-cell subsets (1×10^5^ cells/well) were added to the DCs (100 µl/well) and co-cultured for six days. After 72h, 150 µl of supernatant was harvested and frozen at –80°C for cytokine detection and 150 µl of fresh complete medium was added. The proliferative response was assessed by [^3^H]-thymidine incorporation for the last eight hours of culture followed by scintillation counting.

### Cytokine detection

IFN-γ was detected by ELISA using a sandwich ELISA with mAbs developed against bovine IFN-γ [25]. Flat-bottomed Maxisorp plates (442404, Nunc, Roskilde, Denmark) were coated with the capture anti- IFN-γ CC330 mAb (100 µl/well) (Serotec, Oxford, UK) at 2 µg/ml diluted in PBS and incubated overnight at 4°C. After washes with PBS containing 0.05% Tween20 (PBSTw), wells were saturated with PBSTw supplemented with 5% saccharose and 1% BSA (A9647, Sigma) at room temperature for 60 min. Samples and recombinant IFN-γ diluted in PBSTw supplemented with 1% BSA (dilution buffer) were added (100 µl/well) and incubated at room temperature for 60 min. Washing with PBSTw was repeated between each following step. The anti-IFN-γ:biotin-conjugated CC302 mAb (Serotec) was added (100 µl/well) at 1 µg/ml in dilution buffer and incubated at room temperature for 60 min. Plates were incubated at 37°C for 60 min with horseradish peroxidase-conjugated extravidin (E2886, Sigma-Aldrich) (100 µl/well) diluted at 1/1000 in dilution buffer. Peroxidase activity was revealed by adding tetramethyl benzidine substrate (TMB) (T8665, Sigma). The reaction was stopped by the addition of 0.5M H_2_SO_4_ (50 µl/well) and absorbance was read at 405 nm.

IL-4 detection was similarly performed using anti-bovine CC313 (1 µg/ml) and CC314 (1 µg/ml) mAbs (Serotec) [26]. IL10 was detected by ELISA using the anti-ruminant CC318 and CC320 mAbs (Serotec) as described previously [27].

Standard curves were generated for each cytokine assay with recombinant ovine IFN-γ, IL-4 and IL-10 (kindly provided by Dr S. Wattegedera, MRI, Scotland). Cytokine concentrations were expressed in IU/ml.

### Statistical analyses

In the experiments performed with several sheep, the statistical analysis used was carried out with the package “nparLD” designed to perform non-parametric analysis of longitudinal data in factorial experiments [28]. In the case of the repetition of several independent experiments performed with sorted CD1b^+^ L-DCs and T cells sampled at different times in the same sheep, we have defined that results of proliferative response were representative of a biological response when the ratio of antigen stimulated to medium stimulated conditions was >2.

## Results

### Phenotyping and uptake capacities of migrating CD1b^+^ L-DC at steady state

**Figure 1:**
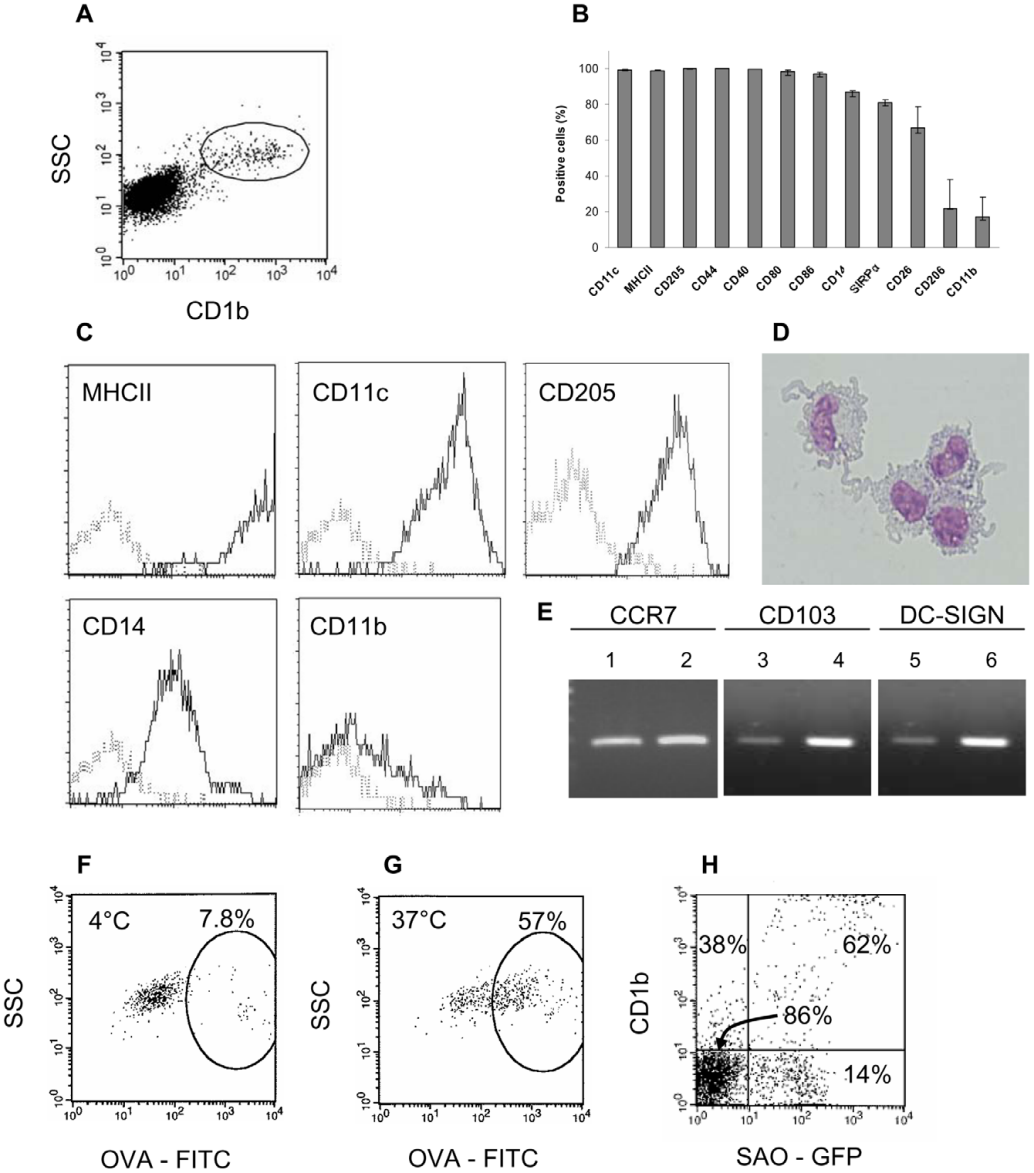
CD1b^+^ L-DCs are migratory mature DC capable of endocytic and phagocytic activities. After labelling lymph cells with anti- CD1b mAb (A), CD1b^+^ L-cells were gated and analysed for the expression of different markers. Data express the median (quartiles) of the percentage of positive cells from 3 sheep (B). Plots of different markers expressed by CD1b^+^ L-DCs are shown (C). May-Grünwald Giemsa staining of sorted and cytocentrifugated CD1b^+^ L-DCs (D). Expression of mRNA for CCR7, CD103 and DC-SIGN by sorted CD1b^+^ L-DCs (1, 3, 5 respectively) and in cDNA control (2, 4, 6 respectively) (E). Uptake of FITC-ovalbumin (FITC-OVA) or GFP-*Salmonella* by CD1b^+^ L- DCs. Lymph cells (1×10^6^ cells) were incubated with FITC-OVA for 1h at 4°C or 37°C, or with GFP-*Salmonella* for 30 min at 37°C, followed by CD1b labelling. CD1b^+^ L- DC subset was gated on lymph cells and FITC-OVA fluorescence shown on dot plots F and G. Total L-APC were gated on SSC/FSC and the cell population negative for lymphocyte markers. Quadrants were then defined on the CD1b^+^ and CD1b^-^ subsets and GFP-*Salmonella* fluorescence shown on dot plot H.

As two subsets of CD1b^+^ L-DCs were found to phagocytose *Salmonella in vitro*, CD1b^+^ CD14^hi^ and CD1b^+^ CD14^lo^ [17], the phenotype of CD1b^+^ L-DCs was characterized on cells in our possession collected from a pseudo-afferent duct draining the skin and to complete further a previous study [29]. CD1b^+^ L-DCs were all found positive for DC-specific markers, i.e., CD11c, MHCII, CD205, CD44 and costimulatory molecules CD40, CD80, CD86, and expressed strongly these molecules (Fig. 1B, 1C). The expression of CD14 on CD1b^+^ L-DCs was positive and relatively homogeneous (Fig. 1C), in contrast to a previous study performed in sheep [18], possibly due to the different sheep breeds used. The clone VPM65 used may recognize a specific CD14 isoform expressed on sheep L-DCs, as clones CAM36 and TUK4 did not label sheep L-DCs [19], [22]. The expression of the other CD26 and SIRP-α markers on the majority of the cells demonstrated the presence of both CD26^+^ and SIRP-α^+^ L- DC subsets in CD1b^+^ L-DCs. Moreover, CD1b^+^ L-DCs expressed CCR7 mRNA as migratory DCs, and CD103 mRNA (Fig. 1E) possibly as dermal DCs, which have been described as CD11c^+^CD11b^lo^CD103^+^ in mice [30], and related to the CD26^+^ L-DC subset which has functional similarities with CD8α-like DCs [17]. In contrast, expression of CD11b and CD206 (Mannose receptor) was observed on less than 20% of the CD1b^+^ L-DCs, and the antibody to human CD209/DC-SIGN did not react with cells, whereas intestinal ovine DCs did [31], and despite the expression of DC-SIGN mRNA (Fig. 1E).

We then investigated the ability of these cells to capture antigens by measuring the endocytic uptake of soluble antigens as FITC-OVA. More than 50% of CD1b^+^ L-DCs were able to endocytose soluble antigens (Fig. 1G). The capacity of CD1b^+^ L-DCs to phagocytose *Salmonella* was also assessed using GFP-*Salmonella* and showed that within the CD1b^+^ L-DC subset, 62% of the cells were fluorescent, whereas only 15% of CD1b^−^ L-DCs were fluorescent (Fig. 1H).

Overall, CD1b^+^ L-DCs have phenotypic features of mature DCs, including those of several conventional DC subsets, and functional abilities to uptake soluble antigens and *Salmonella*.

### CD1b^+^ L-DCs are able to present *Salmonella* antigens to specific effector/memory CD4^+^ T cells

**Figure 2:**
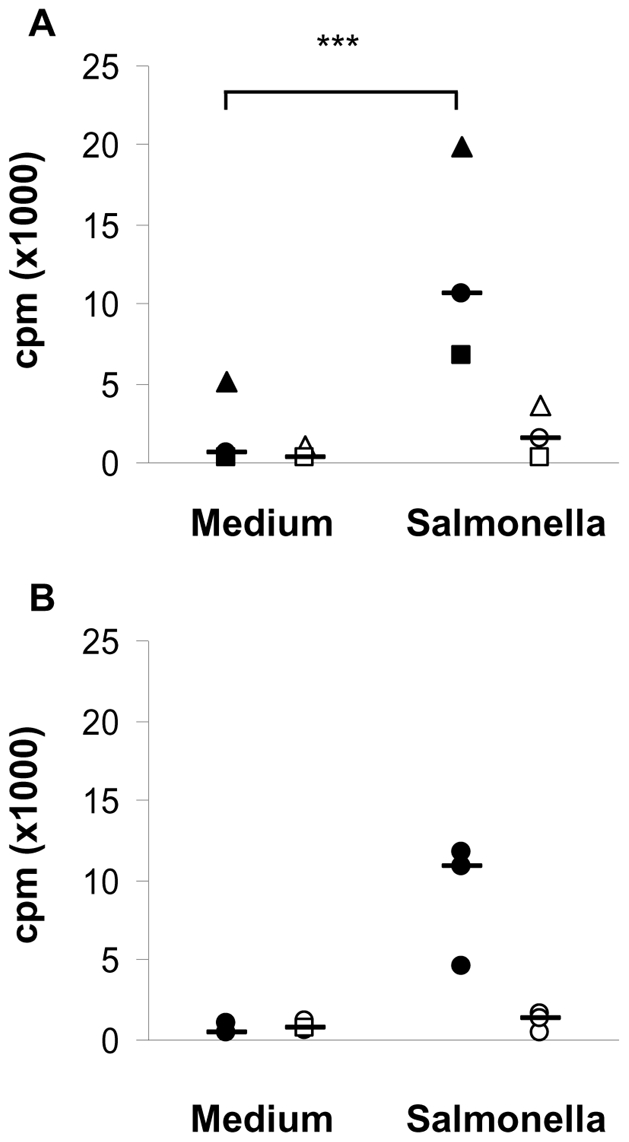
Presentation of *Salmonella* antigens by CD1b^+^ L-DCs to autologous specific CD4^+^ T cells. Sorted CD1b^+^ L-DCs were infected by *Salmonella* for 30 min, incubated for 24h and co-cultured with autologous purified CD4^+^ T cells at a ratio of 1∶100. Cells were co-cultured for six days and the proliferative response assessed by [^3^H]-thymidine incorporation and expressed as counts per minute (cpm). Autologous CD4^+^ (black symbols) or CD4^−^ (white symbols) T cells were either isolated from immune sheep 21 days after SC vaccination with *Salmonella* (A) or from a non-immune sheep (B). Each symbol shape represents data from a different sheep (A) or from three independent experiments performed on one sheep (B). *** very significant difference.

The ability of CD1b^+^ L-DCs to present bacterial antigens to specific T cells, induced *in vivo* by SC infection of sheep by *Salmonella,* was also investigated. To this end, we studied the kinetics of blood T-cell activation following infection. CD4^+^ and CD4^−^ T cells were isolated from the blood of three sheep at different times between one and nine weeks after infection to assess their capacity to be activated by CD1b^+^ L-DCs primed *in vitro* with *Salmonella* (data not shown). The variation in the multiplicity of infection determined the optimal ratio to 100 *Salmonella* per DC to induce the highest proliferative response. The induction of the highest specific proliferative response of CD4^+^ T cells to CD1b^+^ L-DCs primed with *Salmonella* was observed 21 days after infection, and no proliferative response was observed for CD4^−^ T cells (Fig. 2A). Under these conditions, CD1b^+^ L-DCs primed *in vitro* with *Salmonella* were able to activate a high proliferative response (ratio Salmonella/Medium: 21.6) of CD4^+^ T cells and a lower response of CD4^−^ T cells (ratio Salmonella/Medium: 4.9) isolated from non-vaccinated sheep (Fig. 2B). The priming of CD1b^+^ L-DCs with inactivated *Salmonella* induced a lower proliferative response of CD4^+^ T cells than with live *Salmonella* (data not shown). Thus, *in vitro*, CD1b^+^ L-DCs alone are able to phagocytose, process and present efficiently *Salmonella* antigens to effector/memory CD4^+^ T cells.

### CD1b^+^ L-DCs are efficient APC for the priming of naive T cells

**Figure 3:**
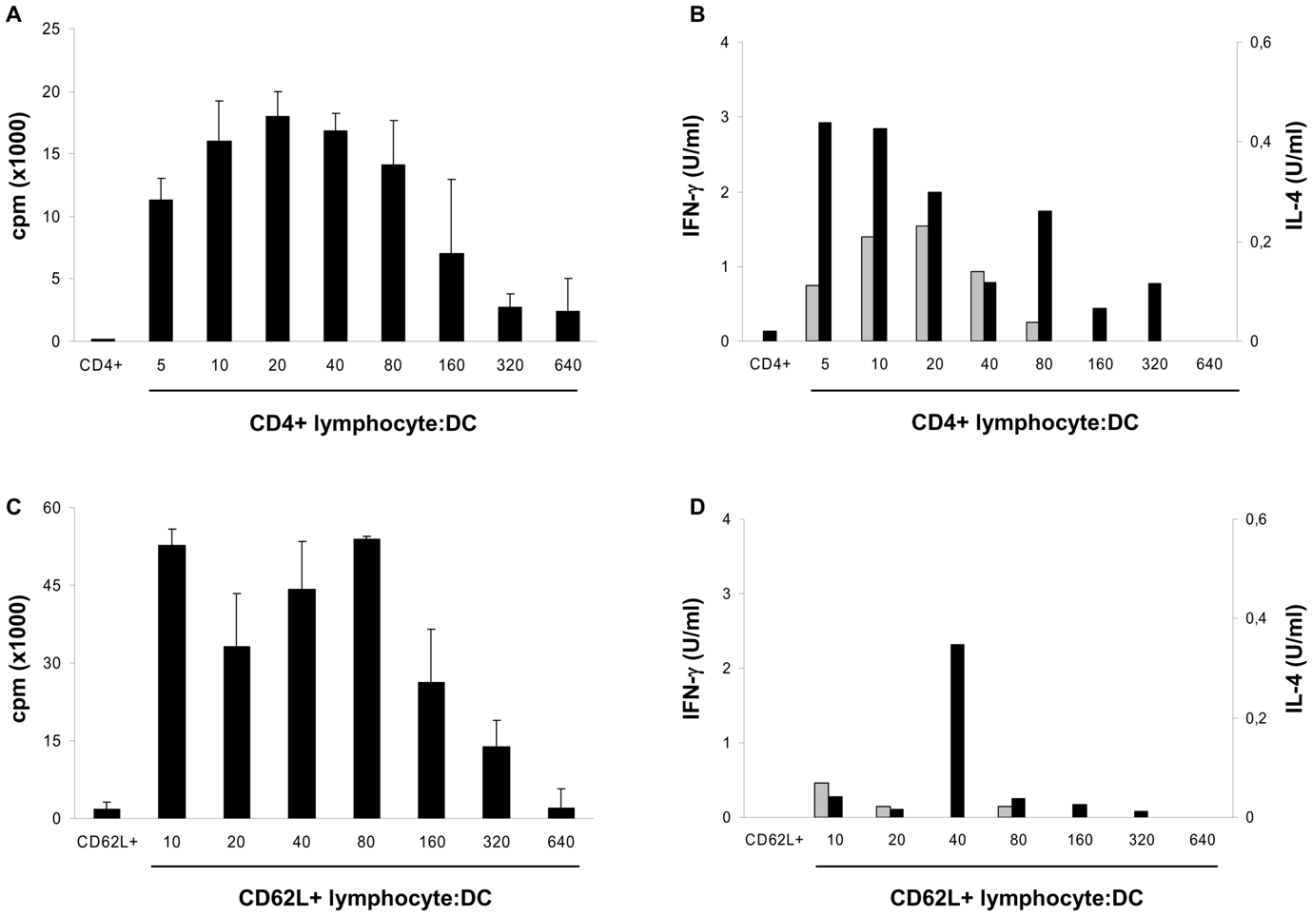
CD1b^+^ L-DCs are efficient APC for the priming of allogeneic naive T cells. Sorted CD1b^+^ L-DCs were incubated at different ratios with 10^5^ allogeneic CD4^+^ T cells (A) or CD62L^+^ T cells (C), and tested for proliferation by [^3^H]-thymidine incorporation after six days of culture. Data express the median (quartiles) of counts per minute (cpm) from triplicates for each condition. After three days of culture, supernatants were sampled and IFN-γ (black bars) and IL-4 (grey bars) production analysed by ELISA (B, D). The results shown are from one representative experiment out of four performed on different sheep (A) or out of two performed on one sheep(C).

The CD1b^+^ L-DCs were then tested for their ability to prime the naive T-cell response. To this end, first an *in vitro* model of allogeneic response was used. Four independent experiments were performed with different sheep and showed that CD1b^+^ L-DCs were able to induce a proliferative response of blood CD4^+^ T cells with an optimal ratio of 20 effector cells per DC (Fig. 3A). This proliferative response was associated with the production of cytokines in the supernatants of CD4^+^ T cell/CD1b^+^ L-DC co-cultures. IFN-γ and IL-4 in a lesser extent, production was detected in supernatants but IL-10 was not (data not shown), demonstrating the predominance of the induced Th1-response (Fig. 3B). CD1b^+^ L-DCs were then tested for their ability to stimulate the priming of allogeneic naive T cells. The naive T cells were sorted by the expression of L-selectin (CD62L), the homing receptor which allows their recruitment into organized lymphoid tissues such as LN via high endothelium venules [32]. CD1b^+^ L-DCs were tested in two allogeneic experiments performed in one sheep with CD62L^+^ T cells. One of these experiments is represented in figure 3C showing the intense proliferative response of CD62L^+^ T cells associated mainly with IFN-γ production (Fig. 3D). Thus, *in vitro*, CD1b^+^ L-DCs alone are able to induce an efficient allogeneic naive T-cell response.

**Figure 4:**
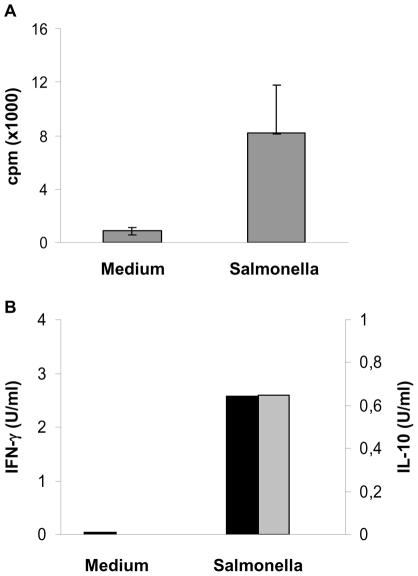
Presentation of *Salmonella* antigens by CD1b^+^ L-DCs to autologous naive CD62L^+^ T cells. Sorted CD1b^+^ L-DCs were infected by *Salmonella* for 30 min, incubated for 24h and co-cultured with autologous purified CD62L^+^ T cells at a ratio of 1∶100. Cells were co-cultured for six days and the proliferative response assessed by [^3^H]-thymidine incorporation (A). Data express the median (quartiles) of counts per minute (cpm) from triplicates for each condition. After three days of culture, supernatants were sampled and IFN-γ (black bars) and IL-10 (grey bars) production analysed by ELISA (B). The results shown are from one representative experiment out of two performed on one sheep.

To evaluate the potential of CD1b^+^ L-DCs to present bacterial antigens to naive T cells, we tested *in vitro* the capacity of CD1b^+^ L-DCs to process and present *Salmonella* to autologous CD62L^+^ T cells. One of the two experiments performed in one sheep is shown in figure 4A and shows a high proliferative response of autologous CD62^+^ T cells (ratio Salmonella/Medium: 12) in co-culture with CD1b^+^ L-DCs infected by live *Salmonella*. This response was associated with the production of both IFN-γ and IL-10, but not IL-4 (Fig. 4B). The CD1b^+^ DCs are thus able to present *Salmonella* antigens to naive T cells efficiently.

### Presentation of *Salmonella* Ag to CD4^+^ T cells by migrating DCs primed by mucosal vaccination with the *Salmonella* Rv-6 strain

**Figure 5:**
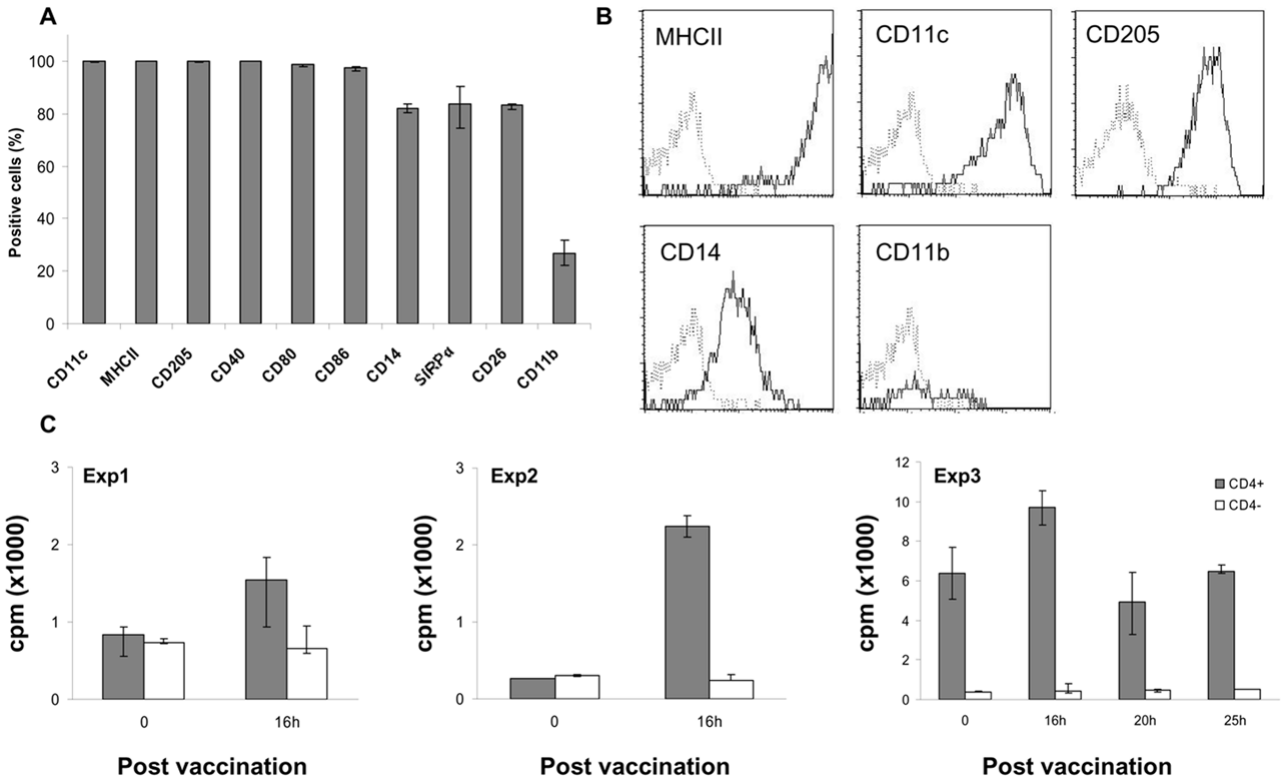
Presentation of *Salmonella* antigens to CD4^+^ T cells by CD1b^+^ L-DCs primed by conjunctival vaccination with the *Salmonella* Rv-6 strain. After labelling of cervical lymph cells with anti- CD1b mAb, CD1b^+^ L-cells were gated and analysed for the expression of different markers. Data express the median (quartiles) of the percentage of positive cells from three sheep (A). Plots of different markers expressed by cervical CD1b^+^ L-DCs are shown (B). Sorted cervical CD1b^+^ L-DCs were isolated from sheep before and after conjunctival vaccination with *Salmonella*, and co-cultured with autologous purified CD4^+^ T cells at a ratio of 1∶100. Cells were co-cultured for six days and the proliferative response assessed by [^3^H]-thymidine incorporation (B). Data express the median (quartiles) of counts per minute (cpm) from triplicates for each condition. The results shown represent three independent experiments performed on one sheep.

To demonstrate further the potential of CD1b^+^ L-DCs to process and present microbial antigens to T cells *in vivo*, the conjunctival route was used to inoculate the *S.* Abortusovis Rv-6 strain vaccine and cannulation of the cervical pseudo-afferent lymph was performed to collect CD1b^+^ L-DCs before and after inoculation. The phenotype of cervical CD1b^+^ L-DCs was analysed at steady state for three sheep, and no difference was observed with the cutaneous CD1b^+^ L-DC phenotype, either in terms of the percentage of cells (Fig. 5A), or of MFI (data not shown). The relative expression of CD103 and DC-SIGN mRNA by cervical CD1b^+^ L-DCs did not differ from that of cutaneous CD1b^+^ L-DCs (data not shown). After inoculation of *Salmonella* by the conjunctival route, the proportion of CD1b^+^ L-DCs did not differ from that before inoculation and the intensity of CD1b expression was similar. After *Salmonella* inoculation, the different marker expression within the CD1b^+^ L-DCs population did not show any significant variation in the percentage of cells or in the MFI (data not shown).

CD1b^+^ L-DCs were then isolated at different times after inoculation to assess their capacity to present *Salmonella* Ag by analysing the proliferative response of autologous CD4^+^ T cells. The results of three independent experiments show a variable proliferative response of CD4^+^ T cells (ratio 16h/0h: 1.8, 8.5, 1.5 respectively), but no response of CD4^−^ T cells, to CD1b^+^ L- DCs collected 16h after inoculation (fig. 5B). This suggests the *in vivo* capture of *Salmonella* antigens by the CD1b^+^ L-DCs, and their capacity to present them directly to CD4^+^ T cells.

## Discussion

This study investigated the potential of migratory DCs to present *Salmonella* Ag directly to T cells in LN, by challenging the CD1b^+^ L-DCs to present Ag of a *Salmonella* vaccine strain to specific and naive T cells using an *in vitro* Ag presentation assay. Although the CD1b^+^ L-DCs display features of mature DCs, they maintain the ability to uptake soluble Ag efficiently and to phagocytose *Salmonella*. Our results show that CD1b^+^ L-DCs alone are able to present *Salmonella* antigens to specific autologous effector/memory CD4^+^ T cells and also to naive T cells associated with a combined IFN-γ and IL-10 response. This was also observed for the priming of allogeneic naive T cells associated with inducing both IFN-γ and IL-4 responses. The potential of CD1b^+^ L-DCs to present *Salmonella* Ag *in vivo* was also shown by collecting CD1b^+^ L-DCs from lymph after conjunctival inoculation of *Salmonella* and by testing their ability to drive the amplification of autologous CD4^+^ T cells.

The migrating CD1b^+^ L-DCs comprising a number of DC subsets could originate from LC, dermal or blood-derived monocytes. At steady state, the high percentage of the CD14^+^ subset in CD1b^+^ L-DCs shows that a large number of L-DCs express the LPS receptor CD14, a monocyte/macrophage-specific molecule, which could testify to the common monocyte-macrophages and DC precursors [33]. The CD26^+^ L-DC subset which has functional similarities with CD8α-like DCs [17], also represents a high proportion of CD1b^+^ L-DCs which could be the cells producing CD103 mRNA and possibly dermal DCs which have been described as CD11c^+^CD11b^lo^CD103^+^ in mice [30]. In contrast, a low percentage of migrating CD1b^+^ L-DCs express CD11b, associated with weak expression of this molecule on the cell surface, suggesting a small proportion of classical dermal DCs within CD1b^+^ L-DCs. Despite the high expression of MHC II and co-stimulatory molecules, CD1b^+^ L-DCs expressed the endocytic receptor as CD206 and showed a good capacity to endocytose soluble Ag. Moreover, 60% of the CD1b^+^ L-DCs were associated with fluorescent *Salmonella,* suggesting the high capacity of CD1b^+^ L-DCs to phagocytose our *Salmonella* vaccine strain. This is in contrast to a previous study showing a limited ability of L-DCs to phagocytose a virulent *Salmonella strain* [18].

Thus, steady-state migrating CD1b^+^ L-DCs exhibit phenotypic features of both mature and immature DCs keeping the ability, on the one hand, to endocytose soluble antigens and phagocytose *Salmonella*, and on the other hand, to express constitutively essential molecules involved in T-cell priming.

Our data showed that CD1b^+^ L-DCs were able to present *Salmonella* antigens to specific effector/memory CD4^+^ T cells efficiently. *In vitro* they were also capable of priming autologous naive T cells to *Salmonella* antigens and of priming allogeneic naive T cells. However, the inactivated vaccine strain amplified CD4^+^ autologous T cells less efficiently than the live vaccine strain. This is in line with a previous study reporting that live virulent *Salmonella* Typhimurium induced a greater up-regulation of the co-stimulatory molecules than killed *Salmonella* [34]. The T-cell subset activated by *Salmonella* clearly comprises the CD4^+^ T cells, and CD1b^+^ L-DCs directed the cytokine responses towards both IFN-γ and IL-10. These results are in line with numerous studies carried out in mice on the immunity to *Salmonella* infection [35] and a few studies performed in sheep [13], [36]. Moreover, CD1b^+^ L-DCs were potentially able to induce IFN-γ and IL-4 responses in the allogeneic reaction. This suggests the potential of these physiologically derived migrating DCs to modulate their response according to the Ag encountered. As a possible Ag delivery vehicle, this *Salmonella* vaccine strain may offer a good tool to drive the immune response towards a balanced IFN-γ and IL-10 response.

To assess the relevance of our data obtained from an *in vitro* model of Ag presentation, we challenged L-DCs collected from sheep inoculated with our *Salmonella* vaccine strain. The phenotype of L-DCs did not change significantly after conjunctival inoculation with the *Salmonella* vaccine strain, in contrast to the increase observed in CD1b and CD14 expression on L-DCs exposed *in vitro* to a virulent strain of *Salmonella* [18]. These differences could be related to the attenuated virulence of the *Salmonella* vaccine strain, but no change was observed in the flow of L-DCs, in contrast to the recruitment of CD1b^+^ L-DCs observed after SC injection of the same strain of *Salmonella* vaccine in oral mucosa [19]. This suggests that the inoculation route could modify the traffic of phagocytes in response to the vaccine strain and to set up the appropriate immune response. In the case of the SC injection, the inflammatory signals induced could trigger the rapid recruitment of granulocytes, monocytes and DCs observed in the lymph [19]. On the other hand, conjunctival administration results in a rapid local control of bacteria by non-specific defences, as previously described with the attenuated Rev1 vaccine strain of *Brucella melitensis* [37], and induces only a minor effect on the lymph traffic with no associated inflammatory response. However, whatever the administration route for the doses used, this vaccine strain is completely cleared in the regional draining LN and is able to induce adaptive protective immunity in pregnant ewes [13]. The CD1b^+^ L-DCs collected after conjunctival administration were able to induce a low, but reproducible, amplification of autologous CD4^+^ T cells, suggesting that these CD1b^+^ L-DCs had processed *in vivo Salmonella* Ag and could present them to T cells. This study did not investigate the processing of *Salmonella* Ag *in vivo*, but we can postulate that the T cells could phagocytose and process *Salmonella* directly from the conjunctiva and/or in the lymph, or acquire Ag from *Salmonella* phagocytosed and killed by granulocytes and monocytes. The main conclusion is that CD1b^+^ L-DCs in our model present potential characteristics of APC to initiate by themselves T-cell priming in the LN, without excluding the involvement of LN-resident DCs. These migrating CD1b^+^ L-DCs are able to uptake, process and present Ag to T cells and to modulate their response according to the Ag encountered. They could be used as target cells for driving immune activation in vaccinal strategies.
